# Opioid Monitoring in Clinical Settings: Strategies and Implications of Tailored Approaches for Therapy

**DOI:** 10.3390/ijms25115925

**Published:** 2024-05-29

**Authors:** Luana M. Rosendo, Tiago Rosado, Thomas Zandonai, Karem Rincon, Ana M. Peiró, Mário Barroso, Eugenia Gallardo

**Affiliations:** 1 Centro de Investigação em Ciências da Saúde, Universidade da Beira Interior (CICS-UBI), 6200-506 Covilhã, Portugal; luanamay.rosendo@hotmail.com; 2 Laboratório de Fármaco-Toxicologia, UBIMedical, Universidade da Beira Interior, EM506, 6200-000 Covilhã, Portugal; 3 Centro Académico Clínico das Beiras (CACB), Grupo de Problemas Relacionados com Toxicofilias, 6200-000 Covilhã, Portugal; 4 Pharmacogenetic Unit, Clinical Pharmacology Department, Alicante Institute for Health and Biomedical Research (ISABIAL), Dr. Balmis General University Hospital, 03010 Alicante, Spain; tzandonai@umh.es (T.Z.); karem.rincong@gmail.com (K.R.); apeiro@umh.es (A.M.P.); 5 Addiction Science Lab, Department of Psychology and Cognitive Science, University of Trento, 38060 Trento, Italy; 6 Department of Pharmacology, Paediatrics and Organic Chemistry, Miguel Hernandez University of Elche, 03550 Alicante, Spain; 7 Clinical Pharmacology Unit, Department of Health of Alicante, University General Hospital Dr. Balmis, 03010 Alicante, Spain; 8 Serviço de Química e Toxicologia Forenses, Instituto Nacional de Medicina Legal e Ciências Forenses-Delegação do Sul, 1169-201 Lisboa, Portugal; mario.j.barroso@inmlcf.mj.pt

**Keywords:** chronic pain, opioids, monitoring, pharmacogenetics, clinical implications

## Abstract

This review emphasises the importance of opioid monitoring in clinical practice and advocates for a personalised approach based on pharmacogenetics. Beyond effectively managing pain, meticulous oversight is required to address concerns about side effects, specially due to opioid-crisis-related abuse and dependence. Various monitoring techniques, along with pharmacogenetic considerations, are critical for personalising treatment and optimising pain relief while reducing misuse and addiction risks. Future perspectives reveal both opportunities and challenges, with advances in analytical technologies holding promise for increasing monitoring efficiency. The integration of pharmacogenetics has the potential to transform pain management by allowing for a precise prediction of drug responses. Nevertheless, challenges such as prominent pharmacogenetic testing and guideline standardisation persist. Collaborative efforts are critical for transforming scientific advances into tangible improvements in patient care. Standardised protocols and interdisciplinary collaboration are required to ensure consistent and evidence-based opioid monitoring. Future research should look into the long-term effects of opioid therapy, as well as the impact of genetic factors on individual responses, to help guide personalised treatment plans and reduce adverse events. Lastly, embracing innovation and collaboration can improve the standard of care in chronic pain management by striking a balance between pain relief and patient safety.

## 1. Introduction

Pain management research faces significant challenges, with significant repercussions for millions of people around the world. Chronic pain, defined as enduring discomfort for more than 3–6 months, affects approximately 20% of the world’s population [1]. This chronic and frequently incapacitating condition affects countless people worldwide, regardless of age, sex, or socioeconomic status. Chronic pain management requires a thorough understanding of its complexities, which include sensory, emotional, cognitive, social, and behavioural aspects. While traditional methods such as medication and physiotherapy are important, they frequently require adaptation to accommodate the unique needs of people suffering from chronic pain. Given its significant impact on both physical and emotional health, there is an ongoing search for truly effective therapeutic approaches [2,3,4].

In the United States, the National Institutes of Health (NIH) reports that over 50 million adults suffer from chronic pain, contributing significantly to healthcare costs and loss of productivity [5,6]. In Europe, studies suggest that chronic pain affects around 20% to 30% of the adult population [7].

According to the Global Burden of Disease Study (GBD 2017), chronic pain is recognised as one of the leading causes of disability worldwide [8]. This study assesses the impact of diseases and injuries globally, providing comprehensive data on the prevalence, severity, and disability associated with various health conditions. Chronic pain can significantly impair individuals’ ability to perform daily activities, work, and engage in social interactions, thereby contributing to its classification as a major contributor to global disability burden [8]. Certain conditions, such as musculoskeletal disorders, arthritis, and neuropathic pain, have a higher incidence on chronic pain situations, especially as populations age [4]. Furthermore, chronic pain disproportionately affects older adults, causing a burden in all aspects of life and frequently resisting traditional treatment approaches.

Opioids, known for their potent pain-relieving characteristics, are essential in pain management, but they come with substantial drawbacks such as adverse responses, tolerance, and dependence. Since their introduction in 1986, opioids have been used as second or third-line therapies for moderate to severe pain, according to the World Health Organization’s (WHO) analgesic ladder. The WHO recently expanded the ladder to four stages, moving opioids from secondary to fourth-line treatment. The use of opioids to treat chronic pain remains consistent with the WHO guidelines [4,9,10,11].

The epidemic opioid crisis highlights the critical need for a more complex approach to opioid therapy. While opioids serve as potent analgesics, their widespread use can exacerbate the crisis. Adopting a uniform approach to opioid therapy that excludes personalised considerations and risk assessments may unintentionally contribute to poor outcomes and increased patient risk. The risks include misuse, addiction, overdose, and adverse reactions, all of which contribute to the ongoing opioid crisis. By recognising the complex interplay of factors contributing to opioid misuse and implementing specific approaches to pain management, healthcare providers can mitigate these risks and work towards addressing the multifaceted challenges of the epidemic opioid crisis [12].

The use of opioids for chronic pain treatment is determined by the patient’s medical history, pain severity, and treatment goals. Common opioids include morphine, codeine, methadone, and hydromorphone, but their use comes with potential risks and side effects; so, healthcare professionals should carefully consider their prescription [11]. Regular monitoring and careful dose adjustments are essential for ensuring the safe and effective use of opioids in chronic pain management. Various pain scales are standardised tools for measuring chronic pain, facilitating healthcare professionals to assess and quantify a patient’s subjective experience of pain. These scales are extremely useful in clinical settings, as they aid in treatment planning and monitoring intervention effectiveness. Pain scales commonly used to assess chronic pain include numeric and visual approaches, such as the Numeric Rating Scale (NRS), Visual Analog Scale (VAS), Verbal Rating Scale (VRS), Faces Pain Scale (FPS), Brief Pain Inventory (BPI), and others [13,14,15]. Notably, NRS and VAS are widely used due to their simplicity and efficacy in quantifying pain intensity [13,14,15]. In contrast, comprehensive questionnaires such as the McGill Pain Questionnaire take a comprehensive approach to pain assessment [15,16]. By using a structured vocabulary to describe the nature, extent, and location of pain, they assess both the sensory and emotional aspects of it. These questionnaires also measure the qualitative aspects of pain and its effects on different aspects of life, in addition to intensity [15,16].

On the other hand, opioid withdrawal syndrome is the collective term for a group of physiological and psychological symptoms that can occur when opioid treatment is stopped or deprescribed. Opioid withdrawal is a serious problem for people interrupting opioid therapy. It can cause symptoms like nausea, vomiting, diarrhoea, muscle aches, anxiety, and insomnia. The type of opioid, dosage, and length of treatment are some of the variables that affect the severity and duration of withdrawal symptoms. For a qualitative evaluation of the withdrawal symptoms severity, clinicians frequently use abstinence or withdrawal scales, such as the Subjective Opiate Withdrawal Scale (SOWS) or the Clinical Opiate Withdrawal Scale (COWS) [17]. These measures provide a consistent framework for assessing withdrawal’s subjective and objective components. Additionally, assessing the potential for addiction, respiratory depression, or gastrointestinal problems is one of the most important secondary effects to consider when evaluating the overall safety and effectiveness of opioid-based chronic pain management. Through the use of qualitative measures, healthcare professionals can better understand how the opioid treatment interruption affects patients and develop individual strategies that decrease withdrawal symptoms and minimise side effects [18].

Introducing personalised medicine earlier in the context of opioid therapy is paramount as it addresses three key blocks: opioids and pain management, pharmacogenetics, and technological innovation in monitoring. Effective pain management is essential, and personalised medicine allows for tailoring opioid therapy to individual needs, optimising pain relief while reducing the risk of adverse effects [19]. Pharmacogenetic insights enable clinicians to predict patient responses to opioids, guiding the selection of the most suitable medication and dosage [10]. Moreover, leveraging technological advancements for real-time monitoring enhances patient safety by providing timely feedback on treatment efficacy and potential adverse reactions, facilitating adjustments to therapy as needed. By integrating personalised medicine at the outset of opioid therapy, healthcare providers can deliver more precise and safer care, ultimately improving patient outcomes and quality of life.

With a focus on the relationship between pharmacogenetics and opioid monitoring, this review article aims to examine the critical role that monitoring opioid use plays among patients prescribed these medications. It seeks to identify important genes that have been thoroughly examined in connection to opioid responses and side effects and synthesise the existing literature. This review seeks to highlight the need for strong monitoring protocols that consider the advantages and disadvantages of opioid therapy. The effectiveness of opioids, identifying side effects, managing opioid dependence, and the wider implications for public health and patient safety are important research areas.

This article’s goal is to provide academics and healthcare professionals with evidence-based insights into how diligent monitoring might improve patient outcomes and inform the development of successful opioid management strategies in clinical practice. It is a narrative review that highlights the available literature and research findings on this topic.

## 2. Pharmacogenomics and Enzyme-Mediated Opioid Activities

Pharmacogenomic (PGx) research serves a variety of important functions in the medical field. These include elucidating the variation in responses observed among clinical study participants and identifying unexpected adverse events (AEs). PGx research helps with the selection of participants for clinical trials, improving study design. Furthermore, it helps to develop diagnostic tests for medications, allowing doctors to identify patients who are more likely to benefit from treatment or are at risk of adverse events. It is worth noting that pharmacogenetics is just one aspect among many others, including age, sex, comorbidities, drug interactions, lifestyle, and socio-economic status, all of which play significant roles.

In addition, PGx research helps to advance our understanding of drug mechanisms, metabolism, disease processes, and individualised dosage requirements. Despite these advances, there is still debate about how genetic findings should be interpreted and integrated into clinical practice [10,20].

Understanding the role of pharmacogenetics with opioids in pain treatment is crucial for optimising therapy, as genotype-guided approaches have been shown to reduce pain intensity, enhance quality of life, and lower opioid prescriptions [21].

Examples of genes that influence drug target receptors and signal transduction pathways include those related to the µ-opioid receptor (*OPRM1*) and to catechol-O-methyltransferase (*COMT*). Aside from *OPRM1*, and *COMT*, several other genes play important roles in opioid PGx. A wide range of genetic variations that influence drug metabolism and elimination processes have been studied. Research in pharmacokinetics has focused on enzymes like those of CYP450 family, glucuronidation enzymes, drug transporter proteins, and the COX enzyme. However, it is worth noting that *CYP2D6*, *OPRM1*, and *COMT* continue to be the focus of research in the literature, reflecting their critical roles in opioid PGx. These genetic variations influence the relationship between drug dose and concentration in the target tissues. Moreover, drug interactions with proteins can be influenced by other medications or patient-related factors, such as sex, age, or pregnancy, as well as environmental factors like diet, exercise, tobacco, and alcohol consumption [10].

From the pharmacogenetics point, the phenotypes of *CYP2D6*, *OPRM1* and *COMT* must follow CPIC guidelines [22].

Individual responses to opioid therapy are significantly influenced by phenotypic variations in key drug metabolism enzymes, particularly *CYP2D6*, *COMT*, and *OPRM1*, with implications for chronic pain management. Understanding enzyme phenotypes is critical for categorising individuals into distinguished metaboliser groups. This diversity in enzymatic activity has global implications for chronic pain patients receiving opioid treatment, emphasising the importance of personalised approaches to improve therapeutic outcomes and reduce potential side effects [10,23,24,25,26].

### 2.1. CYP2D6

The cytochrome P450 2D6 (*CYP2D6*) enzyme, a crucial member of the cytochrome P450 family primarily expressed in the liver but also found in areas of the central nervous system such as substantia nigra, plays a pivotal role in the metabolism of various drugs, including opioids. Responsible for the oxidative metabolism of approximately 25% of commonly prescribed drugs, *CYP2D6* catalyses the biotransformation of opioids into metabolites that can either enhance or diminish the drug’s activity, thereby influencing both its efficacy and potential AEs. Metabolism of opioids occurs in two phases, and can result in the production of both active and inactive metabolites. Active metabolites may have clinical activity and be more effective than the parent drug, or conversely may be associated with higher toxicity [11,26].

Genetic polymorphisms within the *CYP2D6* gene contribute to a broad range of enzymatic activities, resulting in distinct metaboliser phenotypes. Despite constituting only about 2% of all hepatic CYPs, *CYP2D6* metabolises nearly a quarter of important drugs in the human liver [26]. This enzyme has a polymorphism causing significant interindividual and intra-ethnic variability. This leads to over 100 allele variants, which can affect drug clearance and response. These variants affect the expression and function of the corresponding proteins, resulting in significant variability in enzyme activity with therapeutic implications. [26]. These alleles, in various combinations, result in different metabolic activities, spanning from ultra-rapid and extensive metabolisers to intermediate and poor metabolisers [20,26].

Clinical trials have been conducted to investigate the influence of *CYP2D6* polymorphisms on opioid efficacy and adverse effects. These trials often categorise individuals into different phenotypic groups based on their *CYP2D6* activity, such as poor metabolisers (PMs), intermediate metabolisers (IMs), extensive metabolisers (EMs), and ultra-rapid metabolisers (UMs), depending on the specific alleles inherited [27].

The Clinical Pharmacogenetics Implementation Consortium (CPIC) guidelines state that normal metabolisers process opioids at standard rates for expected therapeutic outcomes; intermediate metabolisers metabolise opioids slower than normal but faster than poor metabolisers, requiring potential dosage adjustments for optimal pain relief; extensive metabolisers, processing opioids more rapidly than normal, possibly lead to faster onset of action and increased risk of adverse effects without appropriate dosage adjustments; and rapid metabolisers, akin to ultrarapid metabolisers but without extreme metabolic rates, still require careful monitoring and dosage adjustments for safe and effective pain management [27,28]. Therefore, for instance, CPIC recommends that for poor metabolisers, a switch to a non-opioid analgesic (NSAID) should be considered, or to opioids that are not metabolised by *CYP2D6* (e.g., morphine or hydromorphone), the same occurring with intermediate and ultra-rapid metabolisers [22,24,25,28]

Although *CYP2D6* polymorphism controls the metabolism of some opioids, its action is important for activation of pain management opioids. Indeed, codeine, dihydrocodeine, oxycodone, hydrocodone and tramadol are metabolised by *CYP2D6* [25,26]. Despite the fact that opioids show very weak binding to µ-opioid receptors, O-demethylated metabolites originated by metabolism have more affinity to them, and are therefore more potent than the parent drugs, showing higher analgesic effects [11,22,25].

For instance, in pain management, the concomitant use of prescribed opioids with other analgesics that undergo the CYP450 pathway can lead to drug–drug interactions, affecting both substances’ metabolism. These may be substrates, inhibitors, or inducers of CYP450 and include several clinically prescribed drugs. The same enzyme inhibition may result in opposite effects, either overdose or the patient being nonresponsive to treatment [10,26]. The result will depend on whether the parent opioid is an active compound or a prodrug, respectively. *CYP2D6* inhibitors have the potential to block the effects of the prodrug codeine, which requires oxidation to morphine, in order to produce analgesia. On the other hand, inducers of *CYP2D6* will raise enzymatic activity, leading to a fast elimination of certain opioids (such as hydrocodone), hence decreasing the analgesic benefit, or in the case of codeine, accelerating its conversion to morphine, leading to a possible overdose [10,26].

For codeine and tramadol, the *CYP2D6* enzyme is involved in conversion to active metabolites where the *CYP2D6* activity can be used to predict analgesic effects. The extreme phenotypes (poor and ultra-rapid metaboliser) have a 5–10% prevalence in the White population and have been associated with failure of pain treatment (limited conversion to active metabolites in poor metabolisers) and a higher risk of experiencing adverse events related to painkillers (in ultra-rapid metabolisers) [29,30]. Ultrarapid metabolisers are most often found in the African population [31].

Furthermore, sex differences in opioid response mediated by *CYP2D6* have been observed in clinical studies. Women generally have a higher prevalence of poor *CYP2D6* metaboliser phenotypes compared to men, which can influence their response to opioid therapy. Some research suggests that women may require lower doses of opioids compared to men to achieve similar analgesic effects, possibly due to differences in *CYP2D6* activity and hormonal influences [32,33].

Incorporating pharmacogenetic testing for *CYP2D6* polymorphisms into clinical practice can help optimise opioid therapy by individualising treatment regimens and minimising the risk of adverse events. Further research is needed to elucidate the mechanisms underlying these differences and to develop tailored approaches for pain management based on genetic and biological factors.

### 2.2. COMT

*COMT* is an important enzyme involved in opioid metabolism, particularly relevant for drugs like morphine. Genetic variants in the *COMT* gene result in different enzyme activities, impacting the breakdown of catecholamine neurotransmitters [31,34]. The metabolism of catecholamine neurotransmitters by *COMT* is pivotal in pain response modulation. Catecholamines, encompassing dopamine, epinephrine, and norepinephrine, modulate pain responses by maintaining enkephalin levels in neurons and regulating the activity of the brain’s opioid system [22,25]. Opioids exert their analgesic effects primarily through interaction with opioid receptors in the central nervous system. However, their efficacy and side effects can vary widely among individuals, partly due to differences in opioid metabolism. *COMT*, by metabolising catecholamines, indirectly influences the availability of endogenous opioids, such as endorphins, enkephalins, and dynorphins, which are involved in pain modulation [22,31,34].

Polymorphisms within the *COMT* gene may account for up to 10% of variability in sensitivity to pain, with decreased *COMT* activity resulting in elevated catecholamine levels and heightened pain sensitivity. Conversely, increased *COMT* activity leads to decreased catecholamine levels and reduced pain sensitivity. Dysregulation of dopamine levels due to variations in *COMT* activity may contribute to altered pain sensitivity and response to opioid analgesics [31,34].

*COMT* polymorphisms that affect the rate of catecholamine metabolism have been postulated to play a role in mediating the opioid response. The most frequently studied variant allele, C472G > A (rs4680), encodes a protein with methionine substituted for the wild-type valine. This substitution results in a 3- to 4-fold reduction in enzymatic activity, but conflicting results have been generated in studies assessing the impact of this variant on pain scores and opioid consumption. Individuals homozygous for the wild-type (GG, Val/Val) allele required more morphine than heterozygous or homozygous variant (AA, Met/Met) patients. Somewhat paradoxically, patients homozygous for the variant allele reported higher pain scores but no significant difference in opioid dose requirements for the management of postoperative pain [10,27].

Emerging evidence suggests that sex-specific differences in opioid response may be influenced by variations in the *COMT* gene. Sex hormones, such as oestrogen and testosterone, can modulate *COMT* activity, leading to differential effects on pain processing and opioid sensitivity between men and women [35].

Understanding the interplay between sex-specific factors, *COMT* genetic variations, and opioid response is crucial for optimising pain management strategies and minimising the risk of adverse effects in clinical practice. Further research is warranted to elucidate the underlying mechanisms driving sex-specific differences in opioid sensitivity and to develop personalised approaches to pain management based on individual genetic and hormonal profiles.

### 2.3. OPRM1

The *OPRM1* gene, located on chromosome 6q24-q25, encodes the µ-opioid receptor (MOR), a crucial protein involved in mediating the effects of opioids within the central nervous system. MORs are G protein-coupled receptors primarily expressed in regions of the brain associated with pain modulation, reward processing, and addiction. Upon binding to endogenous opioid peptides like beta-endorphin or exogenous opioid drugs such as morphine, the MOR initiates downstream signalling cascades that result in analgesia, euphoria, and other physiological responses characteristic of opioid action [25,27,31].

Variations in the *OPRM1* gene can influence how opioid receptors respond to opioid agonists, with specific variants like the A118G polymorphism linked to changes in pain perception and the efficacy of opioid treatment. This polymorphism is associated with modifications in opioid binding affinity, receptor signalling, and downstream effects on opioid responsiveness. The presence of wild-type, heterozygous, or homozygous alleles of this gene can impact an individual’s perception of pain and their requirement for opioid therapy. Among the numerous single-nucleotide polymorphisms (SNPs) in the *OPRM1* gene, the A118G variant is the most extensively studied [25,27,31].

The substitution of aspartate (G) for the wild-type asparagine (A) is linked to reduced mRNA and protein expression, suggesting a protective effect of the G allele against pain in chronic pain patients [31]. Women patients with heterozygous or homozygous G alleles demonstrate higher-pressure pain thresholds compared to those with the more common wild-type A allele [31]. However, the correlation between receptor polymorphisms and opioid efficacy remains inconclusive, indicating that different opioids may have varying effects on different alleles [31]. Clinical evidence indicates potential sex-specific differences in opioid sensitivity and response among individuals with distinct *OPRM1* genotypes [35]. Some studies suggest that women carrying the G allele exhibit greater opioid analgesic requirements and increased susceptibility to opioid-induced side effects compared to men with the same genotype, while others report no significant sex differences, emphasising the complexity of sex-related factors and the necessity for further research in this field [31].

Moreover, sex-specific differences in opioid response linked to *OPRM1* genotypes underline the importance of considering biological sex as a variable in personalised pain management approaches. Continued research efforts are needed to elucidate the interplay between genetic, hormonal, and sociocultural factors in shaping opioid outcomes and to develop more effective and personalised strategies for pain relief while minimising the risks associated with opioid therapy.

### 2.4. Clinical Studies

Table 1 presents multiple studies or clinical trials addressing different facets of opioid therapy, such as deprescription, PGx, adverse drug reactions (ADRs), and pain management. It shows that most participants in these studies are Caucasian women. Moreover, oral fluid is the preferred option for genotyping analysis over blood samples.

A few studies examined the effectiveness of long-term opioid deprescription, reporting success rates of approximately 49% in achieving improved pain relief and reduced ADRs [30,35]. In addition, sex differences and pharmacogenetic factors, notably *OPRM1* and *COMT* DNA methylation levels, emerged as significant influencers of deprescription outcomes across the studies. These factors were found to correlate with opioid use disorder, pain relief, quality of life, and adverse events, emphasising their importance in tailoring deprescription strategies [30,36]. On the other hand, when studied, the ultra-rapid metabolisers (*CYP2D6*-UM) consumed three times less basal morphine equivalent daily dose (MEDD), and they showed the highest number of AEs and opioid withdrawal symptoms after discontinuation. This was inversely correlated with their quality of life. Sex differences were evidenced by a tendency towards lower analgesic tolerability in women and lower quality of life in men [37].

Furthermore, genotype-guided treatment approaches demonstrated promising results in reducing pain intensity, enhancing quality of life, and lowering opioid dosages compared to standard prescribing practices. The implementation of multidisciplinary prevention programs for opioid use disorder was highlighted as essential for successful deprescription outcomes, underlining the necessity of collaborative efforts involving healthcare providers [21].

Despite the overarching similarities, variations exist among the studies regarding specific findings and methodologies. For instance, while some studies focused on the association between genotype-guided treatment and opioid dosage reduction [21,38,39,40], others delved into the impact of sex differences and pharmacogenetic factors on deprescription effectiveness [30,37,41]. Variation also arose in the reported prevalence of metabolic phenotypes, with some studies highlighting differences in the distribution of extensive metabolisers (EMs), ultra-rapid metabolisers (UMs), and poor metabolisers (PMs) among the study populations [42].

Moreover, discrepancies were observed in the reported outcomes related to adverse events and treatment tolerability across different studies. While some studies emphasised the role of pharmacogenetic factors in predicting ADRs and treatment efficacy [21,30,35], others focused on the influence of *CYP2D6* polymorphisms on opioid treatment outcomes [28,38,43]. Variability also existed in the observed responses to specific opioids, with differences noted in pain relief, adverse event profiles, and medication tolerability among patient subgroups.

It is significant to mention that just a single article employed PGx and therapeutic drug monitoring to evaluate the clinical efficacy of genotyping chronic pain patients receiving analgesic therapy [42]. This scarcity of studies combining both methods highlights the necessity for additional research in this field. 

**Table 1:** Gene variations and opioids’ pharmacokinetics applied in chronic non-cancer pain (CNCP).

Type of Study	Ethnicity (Number of Individuals)	N (Sex)	Outcome Measured	Gene Assessed	Variant	Findings	Reference
Clinical (Long-term use of opioids deprescription)	n.a.	111 (76 W)	Long-term opioid deprescription	*OPRM1; CYP2D6*	*OPRM1* (rs1799971, 118A > G); *CYP2D6* (*2, *3, *4, *5, *6, *10, *17, *29, *35, *41 and xN)	Long-term opioid deprescription was achieved in 49% of the patients; sex differences and a pharmacogenetic influence were detected.	[30]
Clinical (Controlled study)	Caucasian	50 (40 W)	Effectiveness and safety of PGx-guided opioid therapy	*OPRM1*; *COMT*; *CYP2D6*	*OPRM1* (rs1799971, A118G); *COMT* (rs4680, G472A); *CYP2D6*: *2 (1584C > G), *3 (2550delA), *4 (1847G > A), *5 (*CYP2D6* full gene deletion), *6 (1708delT), *10 (100C > T), *17 (1022C > T), *29 (3184G > A), *35 (31G > A), *41 (2989G > A)	The genotype-guided treatment improved pain relief, quality of life, and reduced AE. It reduced opioid dose by 42% compared to usual prescribing. The final health utility score was higher, improving sleepiness and depression comorbidity, and reducing (30–34%) headache, dry mouth, nervousness, and constipation.	[21]
Retrospective study	Caucasian	250 (125 W)	Sex-mediated genetic–epigenetic interaction	*COMT OPRM1*	*OPRM1*: rs1799971, A118G (A/A; A/G; G/G); *COMT*: rs4680, G472A (G/G; G/A; A/A)	*OPRM1* DNA methylation is linked to lower opioid use disorder cases in women, with patients with lower methylation and the mutant G-allele requiring less opioids. *COMT* DNA methylation levels negatively affect pain relief quality of life and adverse events like constipation, insomnia, and nervousness.	[35]
Start trial	European-American (599); African American (79); Others (96)	764 (240 W)	Effects of variants in 11 genes on dropout rate and dose in patients receiving methadone or buprenorphine/naloxone	*COMT; CYP2D6; OPRM1*	*COMT* (rs4680, G472A); *OPRM1* (rs1799971, A118G); *CYP2D6* Alleles: *1, *3, *4, *5, *6, *7, *8, *9, *41	The pairwise analyses revealed that *COMT* (Val158Met; rs4860) had a nominally significant association with dropout rate in methadone patients.	[36]
Cohort	Caucasian	137 (56 W)	PGx-based changes and recommendations regarding current and potential future medication	*COMT; CYP2D6; OPRM1*	*OPRM1*: A118G rs1799971 *COMT*: rs4633; rs4680; rs4818; rs6269 *CYP2D6*: * 2; *17; *41; *3; *4; *10; *5; *6; *7; *8; *14; *9; *11; *12; *15; *18; *19; *20; *29; *36	PGx variants resulted in clinical recommendations to change PGx-triggering drugs in 33 (32.4%), and other current pharmacotherapy in 23 (22.5%).	[44]
Multicentred study	Caucasian	352 (196 W)	Test whether genotyping may play a role in pain patients clinical setting	*COMT; CYP2D6; OPRM1*	*COMT* 472G > A; *OPRM1* 118A > G, *CYP2D6* Alleles *1, *3, *4, *5, *6, *7, *8, *9, *41	There was a tendency towards increased pain in a gene-dose-dependent manner with the µ-opioid receptor variant *OPRM1* 118A > G.	[38]
Clinical study	Caucasian	172 (128 W)	Sex-based differences	*COMT; OPRM1*	*OPRM1*: rs1799971, A118G (A/A; A/G; G/G); *COMT*: rs4680, G472A (G/G; G/A; A/A)	PGx in a pharmacovigilance recording system enhances understanding of adverse events in CNCP pharmacological therapy, with *OPRM1* and *COMT* polymorphisms linked to gender-specific AEs.	[41]
Cohort (Low back pain)	Caucasian	196 (130 W)	Effectiveness and safety of opioid-based drugs in clinical practice	*CYP2D6*	Allele: *1; *4; *2; *41; *35; *5; *9; *10; *2N; *1N; *6; *15; *35	CYP2D6*6 and *9 carriers, alleles characterised by a reduced (*9) or absent (*6) enzymatic activity were significant.	[43]
Clinical study	Caucasian (54), African American (5), Hispanic (1) and American-Indian (1)	61 (30 W)	Evaluate the clinical effectiveness of genotyping chronic pain patients on analgesic therapy	*CYP2D6*	*3, *4, *5, *6, *7, *8 and gene duplication	Most patients were EMs (54%), followed by IMs (41%), and PMs (5%). Four out of five patients reported ADRs, with 80% having impaired *CYP2D6* metabolism. The only patient with impaired *CYP2D6* metabolism was taking multiple medications partially metabolised by *CYP2D6*.	[42]
Clinical	Caucasian	125 (74 W)	Genotype, inferred phenotype, and urinary and oral fluid codeine O-demethylation metabolites could predict codeine non-response following a short course of codeine	*CYP2D6*	alleles *1, *2, *3, *4, *5, *6, *9, *10, *41	A scoring system was developed to predict analgesic response from day 4 urinary metabolites, with an overall prediction success of 79% for morphine and 79% for the morphine/creatinine ratio, indicating that only 24.5% of normal metabolisers responded to codeine.	[39]
Clinical trial (Comparison study)	Caucasian	584 (415 W)	Effectiveness and security in daily pain practice	*OPRM1 COMT*	*OPRM1*: rs1799971, A118G (A/A; A/G; G/G); *COMT*: rs4680, G472A (G/G; G/A; A/A)	New-generation opioids, OXN and TAP, can control pain intensity but have worse tolerability and higher health resource compared to traditional opioids. COMT genotypes also increase the incidence of some opioid side-effects, especially in women.	[40]
Clinical (Long-term use of opioids deprescription)	n.a.	117 (77 W)	Impact of *CYP2D6* phenotypes and sex on the clinical and safety outcomes	*CYP2D6*	*1, *2, *3, *4, *5, *6, *10, *17, *41, 2D6*5, 2D6 × N, 2D6*4 × 2 gene variants)	*CYP2D6*-UM had three times less basal MEDD and experienced the highest number of adverse events and opioid withdrawal symptoms after deprescription, which was inversely correlated with their quality of life. Sex differences were found to have lower analgesic tolerability in women and lower quality of life in men.	[37]
Clinical trial (Goals vs. Optin)	Asian (3); African American (51); Native or Islander (1); Other (5); Caucasian (162); more than one race (13); unknown (6)	Goals: 125 (70 W) Optin: 119 (55 W)	Patient willingness to consent to PGx testing and the potential for PGx information to support opioid management	*CYP2D6; OPRM1*	*CYP2D6:* *1, *3; *4, *6; *9; *10; *17; *29; *41; *OPRM1*: rs1799971, A118G	The study shows that 55% and 65% of patients are open to pharmacogenetic testing, with 66% and 69% believing it can improve their medical care. It supports the potential of *CYP2D6* PGx testing to inform chronic pain medication management for PMs and UMs.	[45]
Clinical trial	n.a.	370 (252 W)	Effects of *CYP2D6*-guided opioid prescribing on pain control	*CYP2D6*	*CYP2D6*: *1, *2, *3; *4, *5; *6; *7; *8; *9; *10; *11; *15; *17; *29; *35; *41;	The study found that pain intensity among IM/PMs initially prescribed tramadol/codeine showed greater improvement in the *CYP2D6*-guided versus usual care arm, with 24% of *CYP2D6*-guided participants reporting a clinically meaningful reduction. However, no difference in change in composite pain intensity at 3 months was found between *CYP2D6*-guided and usual care groups.	[28]
Clinical trial	Caucasian	88 (56 W)	Prediction of adverse events in prescription opioid use disorder patients	*OPRM1; COMT*	*OPRM1*: rs1799971, A118G (A/A; A/G; G/G); *COMT*: rs4680, G472A (G/G; G/A; A/A)	Wild-type *OPRM1*-AA genotype carriers reported higher adverse events, particularly gastrointestinal system events like nausea. Men had three-times-higher predicted adverse events. The deprescription programme effectively reduced morphine equivalent daily dose and opioid use without affecting pain intensity or opiate abstinence syndrome.	[32]

N: number; n.a.: not available; W: women.

Clinical trials play a vital role in advancing medical knowledge and guiding healthcare practitioners. However, they are subject to various limitations that can impact the interpretation and applicability of their findings. One significant constraint is the reliance on questionnaires for data collection, which introduces subjectivity and may result in incomplete or unreliable information [21,30]. Additionally, the variation among patients in long-term assessments, especially in heterogeneous groups, poses challenges in effectively analysing outcomes [38,41]. Limited sample sizes further hinder the precision and generalisation of findings, potentially compromising statistical power and external validity [21,42]. The retrospective nature of some studies limits data collection and introduces biases, while pragmatic study designs may struggle with blinding and controlling placebo effects [28]. Challenges in assigning phenotypes for genotypes with copy-number variation and limitations in medication assessment further complicate interpretation [41]. Acknowledging and addressing these limitations is crucial for improving the rigor and reliability of clinical trials, ultimately leading to better patient care and outcomes.

In conclusion, while studies collectively underline the significance of sex differences and pharmacogenetic factors in long-term opioid deprescription, variations in methodologies and reported outcomes highlight the complexity of optimising opioid therapy. Despite differences in findings and approaches, the overarching consensus emphasises the importance of personalised medicine approaches, multidisciplinary collaboration, and further research to elucidate the intricate interplay between genetic factors, sex differences, and opioid therapy outcomes.

## 3. Monitoring Opioids

### 3.1. Main Therapeutic Opioid Groups

Opioids are a class of drugs primarily used for their analgesic (pain-relieving) properties [9,18]. However, they have therapeutic applications beyond pain management. The principal groups of therapeutic use for opioids encompass a range of medications that play a vital role in the management of various forms of pain [9,11,18]. Among these, morphine, recognised as one of the oldest and most effective opioids for pain relief, is commonly employed in managing moderate to severe pain, including post-surgical and cancer-related pain. Serving as a prototypical µ-opioid receptor agonist, morphine is considered a cornerstone in the treatment of severe pain, notably in cancer patients [11,46].

Oxycodone, functioning as a µ-opioid receptor agonist, provides an alternative for chronic pain management, particularly when a sustained-release formulation is preferred. This potent opioid is commonly prescribed for chronic pain conditions, available in both immediate-release and extended-release formulations [11,46]. Notably, OxyContin is a widely recognised medication that contains oxycodone in a form that is released slowly into the body over time. Additionally, when combined with acetaminophen or ibuprofen, hydrocodone is a frequently prescribed option for moderate to severe pain, often used in conditions such as osteoarthritis and dental pain [11,46].

Codeine, a less potent opioid, finds application in mild to moderate pain relief and is frequently employed in combination with other medications for its synergistic effects [11,46].

Fentanyl and its derivatives are highly potent synthetic opioids used for short-acting analgesia, particularly in post-surgical or break-through pain. Available in various formulations like patches, lozenges, and injections, they are commonly used for severe pain management, particularly in cancer patients [11,46].

Tramadol, exhibiting a dual mechanism by combining µ-opioid receptor agonism with the inhibition of norepinephrine and serotonin reuptake, provides a unique analgesic profile suitable for moderate to moderately severe pain [11,46].

Methadone, originally acknowledged for its role in opioid addiction treatment, has emerged as a significant player in chronic pain management owing to its extended duration of action and efficacy in addressing neuropathic pain. This long-acting opioid is frequently employed in the treatment of chronic pain, especially in cases where other opioids may not yield optimal responses. Furthermore, methadone is used in opioid rehabilitation for patients with opioid dependence [11,46].

These opioid medications collectively form a diverse pharmacotherapeutic toolkit, allowing healthcare providers to tailor treatment plans to the specific characteristics and needs of individuals experiencing chronic pain. However, it is imperative to approach opioid therapy judiciously, considering not only the potential benefits but also the associated risks, and to continually assess and adapt treatment strategies to optimise outcomes while minimising adverse effects.

### 3.2. Importance of Monitoring

Monitoring opioid therapy is crucial for ensuring safe and effective pain management while minimising the risks associated with opioid use [47].

Monitoring opioid therapy serves multiple crucial purposes in clinical practice. Firstly, it facilitates the assessment of treatment efficacy in managing pain, ensuring that patients receive optimal relief, and allowing for adjustments based on individual responses [48]. Secondly, monitoring aids in the identification and prompt management of potential adverse effects linked to opioids, such as nausea, constipation, sedation, and respiratory depression, thereby enhancing patient safety and comfort. Thirdly, the process helps healthcare providers evaluate the risk of opioid dependence and addiction through regular follow-up appointments, allowing for the early identification of signs indicating escalating use or aberrant behaviours. Moreover, monitoring extends to assessing the impact of pain management on a patient’s daily functioning, including improvements in activities of daily living and overall quality of life. Additionally, it involves the implementation of screening tools to identify signs of opioid misuse or diversion, addressing potential issues early on. Furthermore, monitoring is instrumental in recognising the development of tolerance over time, thereby necessitating dose adjustments to maintain effectiveness. Lastly, ongoing monitoring provides valuable opportunities for patient education, covering the risks and benefits of opioid therapy, proper usage, safe storage, and the importance of compliance with prescribed regimens. Overall, the multifaceted approach of monitoring contributes significantly to optimising opioid therapy outcomes and ensuring patient well-being [48,49,50].

A variety of methodologies and tools are routinely utilised for the comprehensive monitoring of opioid therapy, each serving a distinct role in ensuring the safe and effective management of pain [48,51]. Among these techniques is urine drug testing (UDT), which not only confirms the presence of prescribed opioids but also detects illicit substance use, offering a valuable means of identifying potential misuse or diversion [52]. Additionally, the regular use of pain intensity scales, exemplified by the NRS, coupled with functionality assessments, allows for the quantification and systematic tracking of changes in both pain levels and daily functioning [14,47].

These assessments furnish healthcare professionals with indispensable data for customising interventions to accommodate the progressing requirements of individual patients. Prescription drug monitoring programs (PDMPs) assume a pivotal role by furnishing exhaustive information regarding a patient’s history of controlled substance prescriptions, assisting in the detection of potential instances of misuse [53]. Furthermore, maintaining open communication with patients through regular interviews and self-reports is deemed essential, offering insights into the patient’s holistic experience with pain relief, side effects, and any concerns they may have. Lastly, the inclusion of standardised scales such as the COWS and SOWS proves instrumental in assessing the severity of withdrawal symptoms, providing valuable insights for the judicious management of opioid tapering or discontinuation [17,54]. The seamless integration of these diverse monitoring techniques into clinical practice establishes and maintains a balanced approach to opioid therapy, aiming to optimise pain relief while concurrently minimising the inherent risks associated with prolonged opioid use. This commitment to regular and systematic monitoring not only contributes to patient safety but also facilitates early intervention and supports informed decision-making in the comprehensive management of chronic pain.

### 3.3. Biological Samples Used for Monitoring

The choice of the biological specimen for TM depends on various factors, including the specific requirements of the monitoring program, the desired detection window, and the ease of sample collection [51]. In clinical practice, urine testing remains a common method for routine opioid monitoring due to its convenience, cost-effectiveness, and established reliability [52,55]. Blood and oral fluid testing are used when more immediate information is required, while hair and nail testing are employed for long-term retrospective analyses. Each specimen type has its advantages and limitations, and the selection is often based on the specific goals and context of the opioid monitoring program [55].

For monitoring drug concentrations over time, plasma or serum are often considered the best biological sample, or at least are considered the standard for TDM purposes [51,56]. This is because they provide a clear and accurate representation of the unbound drug concentration in the bloodstream. Monitoring drug levels in plasma allows healthcare professionals to assess the therapeutic efficacy of it, adjust dosage regimens, and minimise the risk of adverse effects. Plasma is preferred for therapeutic drug monitoring because it reflects the portion of the drug that is actively circulating and available for therapeutic action. However, the choice between plasma and serum depends on the specific drug being monitored and the requirements of the assay or analysis method used [51,55].

Oral fluid is a non-invasive and easily collectable biological sample, rendering it suitable for a range of applications. It provides a reflection of the free, unbound drug concentration in the body, making it a valuable indicator for assessing recent drug use. Additionally, oral fluid is useful in evaluating impairment associated with drug presence, offering a convenient and practical method for monitoring drug-related effects [56,57].

Establishing a correlation between plasma (or blood) concentrations and oral fluid concentrations of specific drugs or substances is possible, but the relationship may not be direct and can vary based on the drug’s properties, such as its ability to diffuse from blood into oral fluid. The testing of this specimen offers a non-invasive alternative for monitoring drug levels, particularly in situations where obtaining blood samples is challenging [55,57].

### 3.4. Monitoring Techniques

The combination of extraction and separation techniques with sensitive detection methods allows for accurate monitoring of opioid treatment, ensuring that patients are receiving appropriate doses and minimising the risks associated with opioid therapy. These methods are crucial for therapeutic drug monitoring, assessing adherence to prescribed regimens, and detecting potential misuse or diversion. With regard to the analytical monitoring of opiates, in order to guarantee reliable results, labs must have validated methods in accordance with international guidelines, the most widely used being the FDA and the EMA [3,4].

Miniaturised techniques in analytical chemistry, including microextraction and microscale separation methods, present numerous advantages when compared to conventional approaches like liquid–liquid extraction (LLE) and solid-phase extraction (SPE) (Table 2) [48,56,58,59].

While miniaturised techniques offer numerous advantages, it is essential to note that their successful implementation requires careful consideration of factors such as reproducibility, robustness, and compatibility with specific analytical goals. The choice between miniaturised and conventional techniques depends on the specific requirements of the analysis and the characteristics of the samples being investigated. Table 3 provides a comprehensive summary of techniques employed for the detection and quantification of various opioids.

Despite the equal use of solid-phase extraction (SPE) and liquid–liquid extraction (LLE) in sample extraction applications, a significant difference is evident in microextractions. Only two methods that use solvent-based microextraction (DLLME-SFO and SUPRAS) are observed, whereas the majority of microextractions are based on sorbent-based techniques like magnetic solid phase extraction, MEPS, and SPME. The advantages of sorbent-based microextraction techniques, such as their higher extraction efficiency, lower solvent consumption, and easier operation, can be attributed to this discrepancy. To facilitate effective extraction and concentration, sorbent-based microextraction methods employ materials with a high surface area and specific affinity for target analytes. On the other hand, solvent-based microextraction methods frequently require more exacting optimisation and could be constrained by issues like solvent toxicity and environmental considerations. Sorbent-based microextraction techniques are prevalent, highlighting their adaptability and efficiency in sample preparation for analytical purposes.

Additionally, electrochemical sensors have found application in detection, accounting for approximately 10% of studies.

Urine and plasma are the most common sample types used in opioid analysis, accounting for approximately 64% and 40% of samples, respectively. Blood accounts for 24% of samples, while serum makes up 4%. However, there is increasing interest in alternative sample sources, with oral fluid accounting for 20% and hair for 14% of samples. These alternative samples are gaining popularity due to their potential advantages in opioid testing and monitoring.

The most used analytical technique is high-performance liquid chromatography (HPLC), which is frequently combined with mass spectrometry (MS) or ultraviolet (UV) detection. However, gas chromatography–mass spectrometry (GC-MS), gas chromatography with flame ionisation detection (GC-FID), and other instruments like ion mobility spectrometry (IMS) are also present.

Mass spectrometry (MS) and tandem mass spectrometry (MS/MS) have numerous benefits over other detection techniques such as UV/Vis, DAD (diode array detector), and FID (flame ionisation detector). One of the main advantages is the significant increase in sensitivity [64]. MS and MS/MS detectors can detect analytes at significantly lower concentrations than other methods, making them ideal for trace analysis and compound detection in complex matrices. This higher level of sensitivity is particularly valuable in fields like environmental analysis, research on drugs, and metabolomics, where accurate compound quantification is critical [19,65]. Furthermore, MS and MS/MS have high specificity, which allows for the identification and characterisation of individual compounds within a mixture based on their mass-to-charge ratio. This capability is extremely useful for applications requiring precise compound identification, such as forensic analysis and drug discovery. Overall, the increased sensitivity and specificity provided by MS and MS/MS make them fundamental instruments in analytical chemistry, allowing researchers to improve the accuracy and reliability of their analyses [64].

This review discusses the integration of mass spectrometry with various techniques, including HRMS, QTRAP, and QTOF, among others. Notably, approximately 57% of the instrumentation utilises HPLC, with the most common detectors being UV or MS/MS. In contrast, GC accounts for 30% of the instrumentation, with MS being the most common choice.

The most commonly used method of extraction was LLE, generally combined with liquid or gas chromatography coupled with mass spectrometry.

**Table 3:** Techniques for detection and quantification of opioids.

Compound	Sample (mL)	LOD (ng/mL)	Sample Extraction	Extraction Technique	Instrumentation	Reference
COD, OXY, HYD, TRA	Urine and plasma (200 µL)	Urine: 4.5 for COD; OXY; HYD 7.6 TRA Plasma: 6.1 for COD; OXY; HYD; 9.1 TRA	Extractant solvent: 20 µL HPLC grade water pH = 2; Electric voltage: 400 V	IT-G-EME	HPLC-UV	[66]
TRA	Serum (n.a)	n.a	Electrochemical (DVP)	CoNiWO_4_	Sensor	[67]
MTD, TRA, BUP	Plasma, urine (1 mL)	EME: urine: MTD 6.5; TML 5.0; BUP 8.5; plasma: MTD 50.0; TML 40.0; BUP 75.0 ng/mL; EME-SFME urine: MTD 0.80; TML 0.80; BUP 1.0; plasma: MTD 2.0; TML 2.0; BUP 3.5 ng/mL	EME: electric field of 248 V for 17.5 min and stirring rate at 750 rpm; SFME: 5 µL of 400 mM NaOH added to the acceptor solution (sample solution); organic extract (5 µL of toluene)	EME; EME-SFME	HPLC-UV; CD-IMS	[68]
FNT, ACF, Troc-norfentanyl, Troc-noracetylfentanyl	Urine and plasma (1 mL)	10	LLE extraction 4 mL of solvent for urine and 12 mL for plasma, vortexed at 3000 rpm for 30 s and centrifuged at 5000× *g* for 5 min	LLE (1-clorobutane)	GC-(EI)-MS; HR-LC-(ESI+)-MS	[69]
TRA, COD, MOR, 6-MAM	Blood and urine (1 mL)	10 ng/mL for all except 6-MAM with 5 ng/mL	Extract solvent: 4 mL acetonitrile and 100 mg NaCl; agitated for 10 s; rotation time: 5 min; centrifuged for 3 min at 3500 rpm; extraction time: 35 min	m-d-SPE	GC-(EI)-MS	[70]
MTD	Urine (4 mL) Plasma (1 mL)	n.a.	Plasma: alkalinised using 2 M kalium hydroxide (up to pH 10). Four millilitres of the solvent mixture LLE was added, vortexed for 15 min, and then centrifuged for 10 min at 3400 rpm and 15 °C; urine: alkalinised using 2 M kalium hydroxide (up to pH 10). Ten millilitres of the solvent mixture LLE was added, vortexed for 15 min, and then were centrifuged for 10 min at 3400 rpm and 15 °C	LLE (n-hexane/2-propanol, 97:3, *v*/*v*)	GC-(EI)-MS	[71]
ACF, AF, ISF, VF, 4-FBF, OCF, FAF	Urine (5 mL)	ACF:4.4 ng/L; AF:9.4 ng/L; ISF:3.1 ng/L; VF:5.5 ng/L; 4-FBF:3 ng/L OCF:3.6 ng/L; FAF:9 ng/L	Magnetic biochar (15 mg) was added to sample and shaken for 20 min at a speed of 200 rpm; desorption step: 200 µL methanol for 2 min at 1400 rpm	MSPE	LC-(ESI+)-MS	[72]
MOR	Urine (n.a)	0.58 µM	Electrochemical (SWV)	Fe1W3@CPE	Sensor	[73]
COD, MOR, TRA, OXY	Plasma and urine (1000 μL)	0.5	Conditioning: methanol and water; load: 3×; elution: 200 µL acetonitrile/2-propanol (1:1)	(PT-μSPE)	HPLC-UV	[74]
SUF	Plasma (500 µL)	0.01	LLE for 10 min and centrifuged at 1390× *g*	LLE (2 mL ethyl acetate)	UHPLC-(ESI)-QqQ-MS-MS	[75]
4-ANPP, ACF, AH-7921; ALF, AMF, OHBF, CFN, FNT, FF, isotonitazene; MT-45, DPFF, NMNF, NF, OCF, RF, SUF, TF, U-47700, 6-MAM, BUP, COD, EDDP, EMDP, HYD, HM, MTD, MOR, NC, normorphine, NOR, NOM, OXY, OXM, TRA	Urine (1 mL)	n.a.	The samples were extracted twice with LLE	LLE (1.5 mL chloroform/isopropanol (9:1, *v*/*v*)	GC-(EI)-MS; UHPLC-HRMS (MS2+/−)	[76]
MTD	Plasma, urine, hair (n.a)	0.12 ng/mL	Stirred for 5 min at 70 °C; extraction time: 15 min	HS-SPME	IMS	[77]
MANF, ANF, NF, +/− trans-3-methylnorfentanyl, RF, BCM, VFCM ACF, OCF, BHF, ALF AF, BTF, FNT, 4-ANPP +/− cis-3-methylthiofentanyl, Furanylfentanyl, +/− cis-3-methylfentanyl, para-Fluorofentanyl, ortho-Fluorofentanyl, DPFF, AMF, CFN, Butyrylfentanyl, SUF	Oral fluid (100 uL)	0.05–0.50 ng/mL	Conditioning: 3 times with 250 µL of methanol; 3 times with 250 µL of H2O/methanol/acetonitrile (75:15:10); load: 5 times; washing: 3 times with water; elution: 5 times with 50 µL of methanol 1% HCOOH	MEPS (C_18_)	LC-(ESI+)-HRMS/MS	[78]
TRA	Urine, Oral fluid (n.a.)	9.42 µg/mL	Electrochemical (CV)	Au-SPE/(PANI + AgNPs)/MIP	Sensor	[79]
MOR, COD	Plasma, urine (n.a.)	1.5 ng/mL	pH of the DP: 6.0; membrane composition (agarose concentration: 1% (*w*/*v*) in aqueous media with pH 3.0, and 15 mm thickness); voltage: 25 V; and extraction time: 30 min	G-EME	HPLC-UV	[80]
MOR, OXM, HM, COD OXY, HYD, 6-MAM, ACF, Fentanyl, BEG, TRA, COC, MTD, MPD	Blood (1 mL)	PP: 0.0625–2.5 SPE: 0.125–5	SPE: conditioning: 3 mL of hexane, followed by 3 mL of methanol, and 3 mL of water and 1 mL of 0.1 M phosphate buffer (pH6); washing: 3 mL of water, 2 mL of 0.5 M acetic acid and 3 mL of methanol; elution: 3 mL of dichloromethane/isopropanol/ammonium hydroxide (78:20:2)	PP (2.0 mL of acetonitrile); SPE (200 mg, ZSDAU200 CleanScreen)	LC-(ESI+)-MS/MS	[81]
4-MBF, AF, ALF, CNFDespropionyl-2-fluorofentanyl; 4-ANPP, FNT, FAF, MAF, NF, OCF, RF, SUF	Blood and urine (500 µL)	Blood: 0.01–0.20 Urine: 0.02–0.05	Agitation on a rotating mechanism for 5 min and centrifugation at 2.500× *g* for 5 min	LLE (heptane/isopropyl alcohol/dichloromethane)	UHPL-(ESI+) QTRAP-MS/MS	[82]
MTD; TRA	Urine, plasma, oral fluid (2 mL)	Urine: TRA 0.45; MTD 0.15; Plasma: TRA 2.5; MET 1.2; Oral fluid: TRA 0.8; MTD 0.5	Ultrasonic bath for 5 min; phase separation was done using a magnet.The supernatant was discarded and desorption process was carried out by adding 100 µL acetone to aggregated LDH. Desorption was completed under sonication for 15 min. Magnetic nanoparticles were again separated from the eluent solution by a magnet, the supernatant containing desorbed	UA-MμSPE	GC-MS (n.a)	[83]
NF, ACF, OCF, AF, 4-ANPP, FNT, FAF, AMF, CPF, CFN, butyrfentanyl; 4-FBF	Hair (50 mg)	0.2–1.2 pg/mg	Dichloromethane washed two times and then methanol (1 mL of solvent, vortex mixed for 3 min). The solvent washes were removed following each vortex mixing steps. Following the washing steps, hair was dried at room. One millilitre of methanol was added and the mixture was incubated at 55 °C for 15 h without stirring	n.a.	UHPLC-(ESI+)-QTOF-HRMS	[84]
BUP, COD, FNT, NF, MOR, OXY, TRA, ODT	Hair (50 mg)	n.a.	Sample was treated in an ultrasonic bath for 5 h in 3 mL methanol (50 °C) and allowed to cool down to room temperature; 1.5 mL of the supernatant were evaporated to dryness under nitrogen (55 °C), and reconstituted in 20 µL acetonitrile and 130 µL 2 M ammonium acetate solution	n.a.	LC-(ESI+)-QTRAP-MS	[85]
MTD	Urine, oral fluid, plasma (n.a.)	GC-FID: Urine: 2.5; Plasma 2.7; Oral fluid 9.5 GC-MS: Urine:0.06; Plasma/oral fluid: 0.2	75 µL of 1-undecanol added to the sample; then 500 µL of acetonitrile was added a demulsified; ice-bath for 1 min. The solidified solvent was subsequently transferred to microtube by a spatula and melted at room temperature	DLLME-SFO	GC-FID; GC-MS	[86]
MOR, COD, MTD, TRA, O-TRA,	Blood (100 µL)	5	Centrifuged for 10 min at 14,000 rpm	PP (300 µL methanol)	LC-(ESI+/−)-HRMS	[87]
TRA	Plasma (1 mL); oral fluid (100 µL), urine (10 mL)	Urine and oral fluid:1.5 Plasma: 2.4	20 µL of supramolecular solvent and 20 mg of the sorbent were added into the solution. Air assisted was applied five times in 1 min. Fe_3_O_4_@Cu–Fe –LDH was dispersed thoroughly in the solution and combined with the supramolecular solvent. The sorption of tramadol was accelerated in a short time (1 min) on the surface of the Fe_3_O_4_@Cu–Fe–LDH sorbent. After that, the sorbent was separated from the sample solution by applying a strong magnet (150 × 130 × 50 mm). Subsequently, 100 µL of ethanol was added to elute tramadol from the sorbent by sonication for 1 min. After desorption, the sorbent was isolated from the eluent using a magnet	SUPRAS (1 mL of 1-dodecanol, 3 min of THF); PP (2 mL of acetonitrile to, centrifuged at 2000 rpm for 10 min)	GC-FID	[88]
HYD	Plasma (500 µL) Oral fluid (1 mL)	n.a.	Conditioning: 3 mL methanol and 2 mL of 0.1% TFA; washing: 4 mL of H_2_O/acetonitrile (95:5, *v*/*v*) and 0.1% TFA; elution: 1 mL acetonitrile/H_2_O (80:20, *v*/*v*) and 0.1% TFA	SPE (50 mg, 1 mL Discovery, Supelco)	LC-(ESI)-MS/MS	[89]
MOR	Urine (n.a)	10	n.a.	LLE (n.a.)	SI-MS (EI+)	[90]
COD, MOR, 6-MAM	Blood (250 µL)	5	Conditioning: 3 × 250 µL methanol; 3 × 250 µL 2% formic acid; load: 20 × 250 µL; washing: 1 × 250 µL 3.36% formic acid; elution: 11 × 250 µL 2.36% ammonium hydroxide in methanol.	MEPS (80% C_8_ and 20% SCX)	GC-(ESI+)-MS/MS	[91]
MOR, COD	Blood and urine (1 mL)	0.0018–0.0021	After that, the mixture was placed on the shaker (IKA ^®^ KS 260 basic) for 15 min. Then, the magnetic NC was separated from the sample solution with a forceful magnet (N42 50 × 20; 4123 G). The limpid supernatant solution was decanted after 5 min. Subsequently, the preconcentrated MOR and COD were desorbed from the magnetic adsorbent by using 1 mL of methanol: acetic acid (80: 20 *v*/*v*) solution	MSPE	HPLC-UV-Vis	[92]
TRA, COD, MOR, 6-AC, 6-MAM, FNT	Hair (50 mg)	0.010 TRA, COD, 6-AC; 0.025 MOR; 6-MAM; FNT	Conditioning: 3 × 250 µL of methanol, 3 × 250 µL formic acid 2%; load: 15 × 150 µL; washing: 150 µL of 3.36% formic acid; elution: 8× 100 µL ammonium hydroxide 2.36% in methanol	MEPS (80% C_8_ and 20% SCX)	GC-(ESI+)-MS/MS	[93]
OXY, HYD; FNT, NOR; NH, NF	Urine (10 µL)	40–180 pg/mL	n.a.	n.a.	FSA-CIR	[94]
MOR, HM, COD	Blood (500 µL)	n.a.	Conditioning: 3 mL of methanol, followed by 3 mL of deionised water, and 1 mL of phosphate buffer; washing: 1.5 mL of water, 0.5 mL of 0.1 M acetic acid and 1.5 mL of methanol; elution: 2 mL of ethyl acetate/acetonitrile/ammonium hydroxide (78:20:2)	SPE (130 mg Clean Screen^®^ Dau)	LC-(ESI+)-MS/MS	[95]

4-flubutyryl fentanyl: 4-FBF; 4-flurobutyrfentanyl: 4-FBF; 4-methoxybutyrylfentanyl: 4-MBF; 6-acetylcodeine: 6-AC; 6-Acetylmorphine: 6-MAM; Aµ-SPE: screen-printed gold electrode; Acetylfentanyl: ACF; Acetylnorfentanyl: ANF; Acrylfentanyl: AF; alfa-methylthiofentanyl: BTF; Alfentanil: ALF; Benzoylecgonine: BEG; Beta-hydroxyfentanyl: BHF; Buprenorphine: BUP; Butyrylfentanil Carboxy Metabolite: BCM; Carfentanil: CFN; CD-IMS: Corona discharge ion mobility spectrometry; Cocaine: COC; Codeine: COD; CoNiWO4: cobalt nickel tungstate; CV: cyclic voltammetry; Cyclopropylfentanyl: CPF; Despropionyl para-fluorofentanyl: DPFF; DLLME-SFO: dispersive liquid–liquid microextraction with solidification of floating organic droplet; DPV: differential pulse voltammetry; EI: electron ionisation; EME: electromembrane extraction; ESI-: electrospray ionisation negative; ESI+: electrospray ionisation positive; Fe1W3@CPE: iron tungstate carbon paste electrode; Fentanyl: FNT; FID: Flame ionisation detector; Fluorofentanyl: FF; FSA-CIR: free-solution assay-compensated interferometric reader; Furanylfentanyl: FAF; GC: gas chromatography; G-EME: gel-electromembrane extraction; HPLC: high performance liquid chromatography; HR: high resolution; HRMS: high-resolution mass spectrometry; HS-SPME: headspace solid-phase microextraction; Hydrocodone: HYD; Hydromorphone: HM; IMS: ion mobility spectrometry; Isobutyryl fentanyl: ISF; IT-G-EME: In-tube gel electromembrane extraction; LC: liquid chromatography; LLE: liquid phase extraction; LOD: Limit of detection; m-µSPE: magnetic micro solid extraction; m-d-SPE: magnetic dispersive solid-phase extraction; Meperidine: MPD; MEPS: microextraction by packed sorbent; Methadone: MTD; Methoxyacetyl Norfentanyl: MANF; Methoxyacetylfentanyl: MAF; Morphine: MOR; MS/MS: tandem mass spectrometry; MS: mass spectrometry; MSPE: magnetic solid phase extraction; n.a: not available; N-methyl-norfentanyl: NMNF; Norcodeine: NC; Norfentanyl: NF; Norhydrocodone: NH; Noroxycodone: NOR; Noroxymorphone: NOM; Ocfentanil: OCF; O-desmethyltramadol: ODT; Oxycodone: OXY; Oxymorphone: OXM; PANI + AgNPS/MIP: polyaniline layer coated with silver nanoparticles/molecularly imprinted polymer; PP: protein precipitation; PT-μSPE: Pipette-tip micro solid phase extraction; QqQ: triple quadrupole mass spectrometer; QTOF: quadruple time-of-flight mass spectrometry; QTRAP: quadrupole linear ion trap; Remifentanil: RF; SFME: slug flow microextraction; SI-MS: selected ion monitoring mass spectrometry; SPE: solid phase extraction; Sufentanil: SUF; SUPRAS: supramolecular solvent-based extraction; SWV: square wave voltammetry; Thienyl fentanyl: TF; Trifluoroacetic acid: TFA; Tramadol: TRA; UA-MµSPE: ultrasonic-assisted magnetic solid phase extraction; UHPLC: ultra-high performance liquid chromatography; UV: ultraviolet detector; UV-Vis: ultraviolet-visible detector; Valerylfentanyl: VF; Valerylfentanyl Carboxy Metabolite: VFCM; α-methylfentanyl: AMF; β-hydroxyfentanyl: OHBF.

## 4. Materials and Methods

This study conducted a thorough review of the literature obtained from the PubMed database, with a particular emphasis on pharmacogenetic aspects of chronic pain management and opioid use. Table 1 summarises key genetic factors influencing opioid responses, including “*CYP2D6*”, “*COMT*”, and “*OPRM1*”. This review focused on keywords like “pharmacogenetics”, “chronic pain”, and “opioid use” to explore the genetic factors that influence opioid efficacy, adverse effects, and personalised treatment strategies. Notably, the analysis only included studies involving human subjects, ensuring that the findings are relevant and applicable to clinical practice. Table 2 provides a thorough summary of methods applied for opioid detection and quantification between 2018 to the present. Using search terms like “opioids”, “opiates”, “quantification”, “extraction”, and “biological samples”, this review covered a broad spectrum of cutting-edge opioid analysis techniques and methodologies. This analysis ensured the direct relevance of the techniques to clinical settings by restricting the focus to human studies. This approach facilitated the translation of research findings into improved patient care and the management of conditions related to opioids.

## 5. Conclusions and Future Perspectives

This review highlights the crucial role of opioid monitoring within clinical practice, emphasising a shift towards personalised therapy centred on pharmacogenetics. The necessity for meticulous oversight extends beyond effective pain management to address concerns regarding adverse effects, dependence, and the overarching opioid crisis. Evaluating opioid therapy is paramount for ensuring safe and effective pain management, utilising diverse monitoring techniques and incorporating pharmacogenetic considerations to tailor treatment. Vigilant oversight proves critical in optimising pain relief, addressing concerns of misuse, dependence, and addiction, and improving the precision of opioid therapy through advanced monitoring technologies.

As we contemplate future perspectives in this domain, various opportunities and challenges emerge. Advancements in analytical technologies, including miniaturised techniques and refined mass spectrometric methods, offer promise in enhancing the sensitivity, speed, and cost-effectiveness of opioid monitoring. The potential development of point-of-care testing and wearable devices may revolutionise real-time assessment, providing clinicians with valuable insights for prompt intervention. Furthermore, the integration of pharmacogenetics into opioid monitoring holds significant promise for transforming pain management. Personalised medicine, guided by an individual’s genetic profile, allows for a precise prediction of drug responses, identification of potential adverse events, adjustment of dosage, and the selection of opioids aligned with a patient’s unique pharmacokinetic and pharmacodynamic profile.

Nevertheless, challenges persist, including the imperative for widespread adoption of pharmacogenetic testing in clinical settings, the establishment of standardised guidelines, and the education of healthcare providers on the interpretation of genetic data. Future research should focus on refining the understanding of genetic factors influencing opioid metabolism and response, expanding the repertoire of pharmacogenetic markers, and establishing robust clinical algorithms for incorporating genetic information into treatment decisions.

In the coming years, advancements in technology and a growing body of pharmacogenetic research are poised to enhance the precision and efficacy of individualised opioid therapy. Collaborative efforts among healthcare professionals, geneticists, and researchers will play a pivotal role in translating these scientific advancements into tangible improvements in patient care. Additionally, the combination of artificial intelligence (AI) and big data analytics will transform PGx by allowing for the analysis of large datasets to reveal intricate patterns and correlations. These tools will allow clinicians to make more informed decisions about opioid therapy by taking into account a patient’s genetic makeup, medical history, and other relevant factors. Furthermore, AI algorithms can help predict individual responses to opioids, improving treatment outcomes while reducing the risk of adverse events. Therefore, pharmacogenetics, artificial intelligence, and big data combined are expected to bring about a new era of personalised medicine that will ultimately improve patient safety and well-being. Ultimately, the integration of pharmacogenetics into opioid monitoring heralds a new era in pain management, promising safer and more effective treatment regimens tailored to the unique genetic makeup of each patient.

Within the clinical realm, a pressing need for standardised protocols and guidelines exists to ensure consistent and evidence-based approaches to opioid monitoring. Interdisciplinary collaboration among healthcare providers, pharmacists, and analytical chemists is essential to establish comprehensive frameworks prioritising patient safety and individualised care.

Looking ahead, future research should delve into the long-term effects of opioid therapy, including its impact on patient quality of life, functional outcomes, and the potential for opioid tapering or alternative pain management strategies.

In conclusion, the ongoing evolution of opioid monitoring practices is pivotal in navigating the complex landscape of chronic pain management. Through embracing innovative technologies, refining methodologies, and fostering collaborative efforts, the medical community can aspire to elevate the standard of care for individuals requiring opioid therapy, ultimately achieving a balance between the imperatives of pain relief and patient safety.

## Figures and Tables

**Table 2:** Advantages and disadvantages of miniaturised extraction techniques.

Extraction Technique	Advantage	Disadvantage	Solvent/Sorbent Based	References
PP	Simple, fast and inexpensive	Often requires filtration and centrifugation; limited specificity	Solvent-based	[60]
SPE	High selectivity and concentration capability	Can be time-consuming and uses significant solvent volumes	Sorbent-based	[61]
MEPS	Requires less sample and solvent, automated	Limited capacity for sample loading	Sorbent-based	[48,58]
SPME	Solvent-free, integrates sampling and pre-concentration	Limited to volatile and semi-volatile compounds	Sorbent-based	
µ-SPE	Miniaturised, low solvent use, high throughput	Limited sorbent capacity, potential clogging issues	Sorbent-based	
MSPE	Magnetic separation, easy and fast	Requires magnetic particles, potential for particle loss	Sorbent-based	
LLE	Simple, widely applicable	Uses large amounts of solvent	Solvent-based	[62]
DLLME-SFO	Very low solvent consumption, high enrichment factor	Requires careful handling of the solidified phase	Solvent-based	[63]
SUPRAS	Environmentally friendly, high selectivity	Limited solvent types, sometimes complex preparation	Solvent-based	
EME	High selectivity, low solvent use	Requires specialised equipment, optimal conditions critical	Solvent-based	
IT-G-EME	Enhanced extraction efficiency	Still under research, specifics not widely documented	Solvent-based	
EME-SFME	Combines EME and solid-phase microextraction benefits, high selectivity	Requires complex setup, less widely tested	Solvent-based	
G-EME	Uses greener solvents, potentially more eco-friendly	May involve more complex chemistry, less widely adopted	Solvent-based	

PP: protein precipitation; SPE: solid-phase extraction; MEPS: microextraction by packed sorbent; SPME; solid-phase microextraction; µ-SPE: micro-solid phase extraction; MSPE: magnetic solid phase extraction; LLE: liquid–liquid extraction; DLLME-SFO: dispersive liquid microextraction solid organic floating; SUPRAS: supramolecular solvents; EME: electromembrane extraction; IT-G-EME: in-tube gel electromembrane; SFME: slug flow microextraction; G-EME: gel-electromembrane extraction.

## References

[1] Edinoff A., Sathivadivel N., McBride T., Parker A., Okeagu C., Kaye A.D., Kaye A.M., Kaye J.S., Kaye R.J., Sheth M.M. (2020). Chronic Pain Treatment Strategies in Parkinson’s Disease. Neurol. Int..

[2] Pandelani F.F., Nyalunga S.L.N., Mogotsi M.M., Mkhatshwa V.B. (2023). Chronic pain: Its impact on the quality of life and gender. Front. Pain Res..

[3] Hylands-White N., Duarte R.V., Raphael J.H. (2017). An overview of treatment approaches for chronic pain management. Rheumatol. Int..

[4] Blyth F.M., Briggs A.M., Schneider C.H., Hoy D.G., March L.M. (2019). The Global Burden of Musculoskeletal Pain—Where to From Here?. Am. J. Public Health.

[5] Rikard S.M., Strahan A.E., Schmit K.M., Guy G.P. (2023). Chronic Pain Among Adults—United States, 2019–2021. MMWR. Morb. Mortal. Wkly. Rep..

[6] Buonanno P., Marra A., Iacovazzo C., Vargas M., Nappi S., Squillacioti F., de Siena A.U., Servillo G. (2023). The PATIENT Approach: A New Bundle for the Management of Chronic Pain. J. Pers. Med..

[7] Cohen S.P., Vase L., Hooten W.M. (2021). Chronic pain: An update on burden, best practices, and new advances. Lancet.

[8] James S.L., Abate D., Abate K.H., Abay S.M., Abbafati C., Abbasi N., Abbastabar H., Abd-Allah F., Abdela J., Abdelalim A. (2018). Global, regional, and national incidence, prevalence, and years lived with disability for 354 Diseases and Injuries for 195 countries and territories, 1990-2017: A systematic analysis for the Global Burden of Disease Study 2017. Lancet.

[9] Yang J., Bauer B.A., Wahner-Roedler D.L., Chon T.Y., Xiao L. (2020). The modified WHO analgesic ladder: Is it appropriate for chronic non-cancer pain?. J. Pain Res..

[10] Peiró A.M. (2018). Pharmacogenetics in Pain Treatment. Advances in Pharmacology.

[11] Connors N.J., Mazer-Amirshahi M., Motov S., Kim H.K. (2021). Relative addictive potential of opioid analgesic agents. Pain Manag..

[12] Bonnie R.J., Ford M.A., Phillips J.K. (2017). Pain Management and the Opioid Epidemic.

[13] Atisook R., Euasobhon P., Saengsanon A., Jensen M.P. (2021). Validity and Utility of Four Pain Intensity Measures for Use in International Research. J. Pain Res..

[14] Karcioglu O., Topacoglu H., Dikme O., Dikme O. (2018). A systematic review of the pain scales in adults: Which to use?. Am. J. Emerg. Med..

[15] Hawker G.A., Mian S., Kendzerska T., French M. (2011). Measures of adult pain: Visual Analog Scale for Pain (VAS Pain), Numeric Rating Scale for Pain (NRS Pain), McGill Pain Questionnaire (MPQ), Short-Form McGill Pain Questionnaire (SF-MPQ), Chronic Pain Grade Scale (CPGS), Short Form-36 Bodily Pain Scale (SF-36 BPS), and Measure of Intermittent and Constant Osteoarthritis Pain (ICOAP). Arthritis Care Res..

[16] Robinson C.L., Phung A., Dominguez M., Remotti E., Ricciardelli R., Momah D.U., Wahab S., Kim R.S., Norman M., Zhang E. (2023). Pain Scales: What Are They and What Do They Mean. Curr. Pain Headache Rep..

[17] Wesson D.R., Ling W. (2003). The clinical opiate withdrawal scale (COWS). J. Psychoact. Drugs.

[18] Queremel Milani D.A., Davis D.D. Pain Management Medications. https://www.ncbi.nlm.nih.gov/books/NBK560692/.

[19] Simão A., Antunes M., Gonçalves J., Pires Seixo Soares S., Castro T., Rosado T., Caramelo D., Barroso M., Araujo A., Lopez Rodilla J. (2019). The role of gas chromatography in clinical and forensic toxicology. Gas Chromatography: History, Methods and Applications.

[20] Fredrikson K.M., Fasolino T. (2020). Pharmacogenetic Testing: The Ethics of Implementing in Clinical Practice for Chronic Pain Patients. J. Pain Palliat. Care Pharmacother..

[21] Agulló L., Aguado I., Muriel J., Margarit C., Gómez A., Escorial M., Sánchez A., Fernández A., Peiró A.M. (2023). Pharmacogenetic Guided Opioid Therapy Improves Chronic Pain Outcomes and Comorbid Mental Health: A Randomized, Double-Blind, Controlled Study. Int. J. Mol. Sci..

[22] Crews K.R., Monte A.A., Huddart R., Caudle K.E., Kharasch E.D., Gaedigk A., Dunnenberger H.M., Leeder J.S., Callaghan J.T., Samer C.F. (2021). Clinical Pharmacogenetics Implementation Consortium Guideline for *CYP2D6*, *OPRM1*, and *COMT* Genotypes and Select Opioid Therapy. Clin. Pharmacol. Ther..

[23] Magarbeh L., Gorbovskaya I., Le Foll B., Jhirad R., Müller D.J. (2021). Reviewing pharmacogenetics to advance precision medicine for opioids. Biomed. Pharmacother..

[24] Hicks J., Bishop J., Sangkuhl K., Müller D., Ji Y., Leckband S., Leeder J., Graham R., Chiulli D., LLerena A. (2015). Clinical Pharmacogenetics Implementation Consortium (CPIC) Guideline for *CYP2D6* and *CYP2C19* Genotypes and Dosing of Selective Serotonin Reuptake Inhibitors. Clin. Pharmacol. Ther..

[25] Ballantyne J.C. (2006). Opioid Therapy. ASA Refresh. Courses Anesthesiol..

[26] Antunes M., Simão A.Y., Fonseca S., Gonçalves J., Soares S., Almeida E., Gameiro C., Rosado T., Duarte A.P., Barroso M. (2020). The role of *CYP2D6* in opiates and opioids consumers and its implications in clinical and forensic toxicology. Advances in Medicine and Biology.

[27] Nerenz R.D., Tsongalis G.J. (2018). Pharmacogenetics of Opioid Use and Implications for Pain Management. J. Appl. Lab. Med..

[28] Smith D.M., Weitzel K.W., Elsey A.R., Langaee T., Gong Y., Wake D.T., Duong B.Q., Hagen M., Harle C.A., Mercado E. (2019). *CYP2D6*-guided opioid therapy improves pain control in *CYP2D6* intermediate and poor metabolizers: A pragmatic clinical trial. Genet. Med..

[29] Ruaño G., Larsen K., Kocherla M., Graydon J.S., Kost J. (2017). Complications of psychotropic and pain medications in an ultrarapid metabolizer patient at the upper 1% of Cytochrome P450 (CYP450) function quantified by combinatorial CYP450 genotyping. J. Pain Palliat. Care Pharmacother..

[30] Muriel J., Escorial M., Margarit C., Barrachina J., Carvajal C., Morales D., Peiró A.M. (2023). Long-term deprescription in chronic pain and opioid use disorder patients: Pharmacogenetic and sex differences. Acta Pharm..

[31] Cornett E.M., Carroll Turpin M.A., Pinner A., Thakur P., Sekaran T.S.G., Siddaiah H., Rivas J., Yates A., Huang G.J., Senthil A. (2020). Pharmacogenomics of Pain Management: The Impact of Specific Biological Polymorphisms on Drugs and Metabolism. Curr. Oncol. Rep..

[32] Muriel J., Margarit C., Barrachina J., Ballester P., Flor A., Morales D., Horga J.F., Fernández E., Peiró A.M. (2019). Pharmacogenetics and prediction of adverse events in prescription opioid use disorder patients. Basic Clin. Pharmacol. Toxicol..

[33] Lopes G.S., Bielinski S.J., Moyer A.M., Black J.L., Jacobson D.J., Jiang R., Larson N.B., Sauver J.L.S. (2020). Sex Differences in Associations Between *CYP2D6* Phenotypes and Response to Opioid Analgesics. Pharmgenomics. Pers. Med..

[34] Vetterlein A., Monzel M., Reuter M. (2023). Are catechol-O-methyltransferase gene polymorphisms genetic markers for pain sensitivity after all?—A review and meta-analysis. Neurosci. Biobehav. Rev..

[35] Agulló L., Muriel J., Margarit C., Escorial M., Garcia D., Herrero M.J., Hervás D., Sandoval J., Peiró A.M. (2023). Sex Differences in Opioid Response Linked to OPRM1 and COMT genes DNA Methylation/Genotypes Changes in Patients with Chronic Pain. J. Clin. Med..

[36] Crist R.C., Li J., Doyle G.A., Gilbert A., Dechairo B.M., Berrettini W.H. (2018). Pharmacogenetic analysis of opioid dependence treatment dose and dropout rate. Am. J. Drug Alcohol Abus..

[37] Muriel J., Barrachina J., Del Barco G., Carvajal C., Escorial M., Margarit C., Ballester P., Peiró A.M. (2023). Impact of *CYP2D6* genotype on opioid use disorder deprescription: An observational prospective study in chronic pain with sex-differences. Front. Pharmacol..

[38] Lötsch J., von Hentig N., Freynhagen R., Griessinger N., Zimmermann M., Doehring A., Rohrbacher M., Sittl R., Geisslinger G. (2009). Cross-sectional analysis of the influence of currently known pharmacogenetic modulators on opioid therapy in outpatient pain centers. Pharmacogenet. Genom..

[39] Radford H., Simpson K.H., Rogerson S., Johnson M.I. (2019). A single site population study to investigate *CYP2D6* phenotype of patients with persistent non-malignant pain. Medicina.

[40] Barrachina J., Margarit C., Muriel J., López-Gil S., López-Gil V., Vara-González A., Planelles B., del Inda M.-M.M., Morales D., Peiró A.M. (2022). Oxycodone/naloxone versus tapentadol in real-world chronic non-cancer pain management: An observational and pharmacogenetic study. Sci. Rep..

[41] Planelles B., Margarit C., Inda M.-M., Ballester P., Muriel J., Barrachina J., Ajo R., Esteban M.-D., Peiró A.M. (2020). Gender based differences, pharmacogenetics and adverse events in chronic pain management. Pharmacogenomics J..

[42] Jannetto P.J., Bratanow N.C. (2009). Utilization of pharmacogenomics and therapeutic drug monitoring for opioid pain management. Pharmacogenomics.

[43] Dagostino C., Allegri M., Napolioni V., D’agnelli S., Bignami E., Mutti A., van Schaik R.H.N. (2018). *CYP2D6* genotype can help to predict effectiveness and safety during opioid treatment for chronic low back pain: Results from a retrospective study in an italian cohort. Pharmgenomics. Pers. Med..

[44] Niedrig D.F., Rahmany A., Heib K., Hatz K.-D., Ludin K., Burden A.M., Béchir M., Serra A., Russmann S. (2021). Clinical Relevance of a 16-Gene Pharmacogenetic Panel Test for Medication Management in a Cohort of 135 Patients. J. Clin. Med..

[45] Kusic D., Heil J., Zajic S., Brangan A., Dairo O., Smith G., Morales-Scheihing D., Buono R.J., Ferraro T.N., Haroz R. (2022). Patient Perceptions and Potential Utility of Pharmacogenetic Testing in Chronic Pain Management and Opioid Use Disorder in the Camden Opioid Research Initiative. Pharmaceutics.

[46] Morrone L.A., Scuteri D., Rombolà L., Mizoguchi H., Bagetta G. (2017). Opioids Resistance in Chronic Pain Management. Curr. Neuropharmacol..

[47] Larochelle M.R., Lodi S., Yan S., Clothier B.A., Goldsmith E.S., Bohnert A.S.B. (2022). Comparative Effectiveness of Opioid Tapering or Abrupt Discontinuation vs. No Dosage Change for Opioid Overdose or Suicide for Patients Receiving Stable Long-term Opioid Therapy. JAMA Netw. Open.

[48] Soares S., Rosado T., Barroso M., Gallardo E. (2023). Solid Phase-Based Microextraction Techniques in Therapeutic Drug Monitoring. Pharmaceutics.

[49] Oellerich M. (1996). International Association of Therapeutic Drug Monitoring and Clinical Toxicology: Newsletter. Ther. Drug Monit..

[50] De Rosa F., Giannatiempo B., Charlier B., Coglianese A., Mensitieri F., Gaudino G., Cozzolino A., Filippelli A., Piazza O., Dal Piaz F. (2023). Pharmacological Treatments and Therapeutic Drug Monitoring in Patients with Chronic Pain. Pharmaceutics.

[51] Adaway J.E., Keevil B.G. (2012). Therapeutic drug monitoring and LC–MS/MS. J. Chromatogr. B.

[52] SAMHSA (2012). Clinical Drug Testing in Primary Care: Technical Assistance Publication Series TAP 32.

[53] Gudoski A.J. (2015). Prescription drug monitoring programs: Combating prescription drug misuse. Nurse Pract..

[54] Validity of Outcome Measures-Clinical Review Report: Buprenorphine Extended-Release Injection (Sublocade)-NCBI Bookshelf. https://www.ncbi.nlm.nih.gov/books/NBK546449/.

[55] Avataneo V., D’Avolio A., Cusato J., Cantù M., De Nicolò A. (2019). LC-MS application for therapeutic drug monitoring in alternative matrices. J. Pharm. Biomed. Anal..

[56] Agrawal A., Keçili R., Ghorbani-Bidkorbeh F., Hussain C.M. (2021). Green miniaturized technologies in analytical and bioanalytical chemistry. TrAC Trends Anal. Chem..

[57] Gallardo E., Rosado T., Barroso M. (2023). The potential of oral fluid in drug monitoring: An update. Bioanalysis.

[58] Rosendo L.M., Brinca A.T., Pires B., Catarro G., Rosado T., Guiné R.P.F., Araújo A.R.T.S., Anjos O., Gallardo E. (2023). Miniaturized Solid Phase Extraction Techniques Applied to Natural Products. Processes.

[59] Sajid M., Płotka-Wasylka J. (2022). Green analytical chemistry metrics: A review. Talanta.

[60] Krishna M.V., Padmalatha K., Madhavi G. (2021). Electromembrane Extraction in Analytical Toxicology. Microextraction Tech. Anal. Toxicol..

[61] Poole C.F., Poole S.K. (2012). Principles and Practice of Solid-Phase Extraction. Compr. Sampl. Sample Prep. Anal. Tech. Sci..

[62] Kyle P.B. (2017). Toxicology: GCMS. Mass Spectrometry for the Clinical Laboratory.

[63] Bayatloo M.R., Tabani H., Nojavan S., Alexovič M., Ozkan S.A., Alexovi M. (2023). Liquid-Phase Microextraction Approaches for Preconcentration and Analysis of Chiral Compounds: A Review on Current Advances. Crit. Rev. Anal. Chem..

[64] Li C., Chu S., Tan S., Yin X., Jiang Y., Dai X., Gong X., Fang X., Tian D. (2021). Towards Higher Sensitivity of Mass Spectrometry: A Perspective From the Mass Analyzers. Front. Chem..

[65] Rosado T., Soares S., Malaca S., Gonçalves J., Barroso M., Gallardo E., Lucero I. (2018). The role of liquid chromatography in toxicological analysis. High-Performance Liquid Chromatography: Types, Parameters and Applications.

[66] Rahbarian H., Nojavan S., Maghsoudi M., Tabani H. (2023). In-tube gel electromembrane extraction: A green strategy for the extraction of narcotic drugs from biological samples. J. Chromatogr. A.

[67] Zafar K., Wasim M., Fatima B., Hussain D., Mehmood R., Najam-ul-Haq M. (2023). Quantification of tramadol and serotonin by cobalt nickel tungstate in real biological samples to evaluate the effect of analgesic drugs on neurotransmitters. Sci. Rep..

[68] Behpour M., Maghsoudi M., Nojavan S. (2022). Analysis of methamphetamine, methadone, tramadol, and buprenorphine in biological samples by ion mobility spectrometry after electromembrane extraction in tandem with slug flow microextraction. J. Chromatogr. A.

[69] Valdez C.A., Leif R.N., Corzett T.H., Dreyer M.L. (2022). Analysis, identification and confirmation of synthetic opioids using chloroformate chemistry: Retrospective detection of fentanyl and acetylfentanyl in urine and plasma samples by EI-GC-MS and HR-LC-MS. PLoS ONE.

[70] Yasien S., Ali E., Javed M., Iqbal M.M., Iqbal S., Alrbyawi H., Aljazzar S.O., Elkaeed E.B., Dera A.A., Pashameah R.A. (2022). Simultaneous Quantification of Opioids in Blood and Urine by Gas Chromatography-Mass Spectrometer with Modified Dispersive Solid-Phase Extraction Technique. Molecules.

[71] Ciucă Anghel D.M., Ciobanu A.M., Guțu C.M., Stan M., Tudor G., Baconi D.L. (2022). GC-MS Analysis of Methadone and EDDP in Addicted Patients under Methadone Substitution Treatment: Comparison of Urine and Plasma as Biological Samples. Molecules.

[72] Zhang S., Hua Z., Yao W., Lü T., Zhang D., Zhao Q., Li J., Zhao H. (2022). Preparation of bamboo-derived magnetic biochar for solid-phase microextraction of fentanyls from urine. J. Sep. Sci..

[73] Ognjanović M., Nikolić K., Bošković M., Pastor F., Popov N., Marciuš M., Krehula S., Antić B., Stanković D.M. (2022). Electrochemical Determination of Morphine in Urine Samples by Tailoring FeWO4/CPE Sensor. Biosensors.

[74] Hejabri Kandeh S., Amini S., Ebrahimzadeh H. (2021). Simultaneous trace-level monitoring of seven opioid analgesic drugs in biological samples by pipette-tip micro solid phase extraction based on PVA-PAA/CNT-CNC composite nanofibers followed by HPLC-UV analysis. Microchim. Acta.

[75] Zawadzki M., Kowalski G., Chłopaś-Konowałek A., Siczek M., Sobieszczańska M., Leppert W., Wieczorowska-Tobis K., Szpot P. (2021). Rapid Determination of Sufentanil in Human Plasma by UHPLC–QqQ-MS-MS. J. Anal. Toxicol..

[76] Marchei E., Ferri M.A., Torrens M., Farré M., Pacifici R., Pichini S., Pellegrini M. (2021). Ultra-High Performance Liquid Chromatography-High Resolution Mass Spectrometry and High-Sensitivity Gas Chromatography-Mass Spectrometry Screening of Classic Drugs and New Psychoactive Substances and Metabolites in Urine of Consumers. Int. J. Mol. Sci..

[77] Abedi H. (2021). Solid-phase microextraction of methadone by using a chitosan nanocomposite incorporated with Polyoxomolibdate nanocluster/Graphene oxide. J. Sep. Sci..

[78] Vincenti F., Montesano C., Pirau S., Gregori A., Di Rosa F., Curini R., Sergi M. (2021). Simultaneous quantification of 25 fentanyl derivatives and metabolites in oral fluid by means of microextraction on packed sorbent and lc–hrms/ms analysis. Molecules.

[79] Diouf A., Aghoutane Y., Burhan H., Sen F., Bouchikhi B., El Bari N. (2021). Tramadol sensing in non-invasive biological fluids using a voltammetric electronic tongue and an electrochemical sensor based on biomimetic recognition. Int. J. Pharm..

[80] Rahimi A., Nojavan S., Tabani H. (2020). Inside gel electromembrane extraction: A novel green methodology for the extraction of morphine and codeine from human biological fluids. J. Pharm. Biomed. Anal..

[81] Wagner R., Moses L. (2021). Validation of two methods for the quantitative analysis of cocaine and opioids in biological matrices using LCMSMS. J. Forensic Sci..

[82] Jung J., Kolodziej A., Pape E., Bisch M., Javot L., Gibaja V., Jouzeau J.-Y., Scala-Bertola J., Gambier N. (2020). Multiplex detection of 14 fentanyl analogues and U-47700 in biological samples: Application to a panel of French hospitalized patients. Forensic Sci. Int..

[83] Adlnasab L., Shahdousti P., Ahmar H. (2020). Layered double hydroxide intercalated with tyrosine for ultrasonic-assisted microextraction of tramadol and methadone from biological samples followed by GC/MS analysis. Microchim. Acta.

[84] Salomone A., Di Corcia D., Negri P., Kolia M., Amante E., Gerace E., Vincenti M. (2021). Targeted and untargeted detection of fentanyl analogues and their metabolites in hair by means of UHPLC-QTOF-HRMS. Anal. Bioanal. Chem..

[85] Musshoff F., Schwarz G., Sachs H., Skopp G., Franz T. (2020). Concentration distribution of more than 100 drugs and metabolites in forensic hair samples. Int. J. Legal Med..

[86] Jafarinejad M., Ezoddin M., Lamei N., Abdi K., Babhadi-Ashar N., Pirooznia N., Akhgari M. (2020). Effervescent tablet-assisted demulsified dispersive liquid–liquid microextraction based on solidification of floating organic droplet for determination of methadone in water and biological samples prior to GC-flame ionization and GC-MS. J. Sep. Sci..

[87] Joye T., Rocher K., Déglon J., Sidibé J., Favrat B., Augsburger M., Thomas A. (2020). Driving Under the Influence of Drugs: A Single Parallel Monitoring-Based Quantification Approach on Whole Blood. Front. Chem..

[88] Ezoddin M., Adlnasab L., Afshari Kaveh A., Karimi M.A., Mahjoob B. (2019). Development of air-assisted dispersive micro-solid-phase extraction-based supramolecular solvent-mediated Fe3O4@Cu–Fe–LDH for the determination of tramadol in biological samples. Biomed. Chromatogr..

[89] Deatherage Kaiser B.L., Jacobs J.M., Schepmoes A.A., Brewer H.M., Webb-Robertson B.J.M., Valtier S., Bebarta V.S., Adkins J.N. (2020). Assessment of the Utility of the Oral Fluid and Plasma Proteomes for Hydrocodone Exposure. J. Med. Toxicol..

[90] Usmanov D.T., Akhunov S.D., Khasanov U., Rotshteyn V.M., Kasimov B.S. (2020). Direct detection of morphine in human urine by surface-ionization mass spectrometry. Eur. J. Mass Spectrom..

[91] Prata M., Ribeiro A., Figueirinha D., Rosado T., Oppolzer D., Restolho J., Araújo A.R.T.S., Costa S., Barroso M., Gallardo E. (2019). Determination of opiates in whole blood using microextraction by packed sorbent and gas chromatography-tandem mass spectrometry. J. Chromatogr. A.

[92] Abdolmohammad-Zadeh H., Zamani A., Shamsi Z. (2019). Preconcentration of morphine and codeine using a magnetite/reduced graphene oxide/silver nano-composite and their determination by high-performance liquid chromatography. J. Chromatogr. A.

[93] Rosado T., Barroso M., Vieira D.N., Gallardo E. (2019). Determination of Selected Opiates in Hair Samples Using Microextraction by Packed Sorbent: A New Approach for Sample Clean-up. J. Anal. Toxicol..

[94] Kammer M.N., Kussrow A., Gandhi I., Drabek R., Batchelor R.H., Jackson G.W., Bornhop D.J. (2019). Quantification of Opioids in Urine Using an Aptamer-Based Free-Solution Assay. Anal. Chem..

[95] Cannaert A., Vasudevan L., Friscia M., Mohr A.L.A., Wille S.M.R., Stove C.P. (2018). Activity-Based Concept to Screen Biological Matrices for Opiates and (Synthetic) Opioids. Clin. Chem..

